# The Embodied Self, the Pattern Theory of Self, and the Predictive Mind

**DOI:** 10.3389/fpsyg.2018.02270

**Published:** 2018-11-23

**Authors:** Albert Newen

**Affiliations:** Institut für Philosophie II, Ruhr-Universität Bochum, Bochum, Germany

**Keywords:** self, embodied self, predictive coding, pattern theory, self-model

## Abstract

Do we have to presuppose a self to account for human self-consciousness? If so, how should we characterize the self? These questions are discussed in the context of two alternatives, i.e., the no-self position held by Metzinger (2003, 2009) and the claim that the only self we have to presuppose is a narrative self (Dennett, 1992; Schechtman, 2007; Hardcastle, 2008) which is primarily an abstract entity. In contrast to these theories, I argue that we have to presuppose an embodied self, although this is not a metaphysical substance, nor an entity for which stable necessary and jointly sufficient conditions can be given. Self-consciousness results from an integration of an embodied, basic affective flow with an intentional object (the self as agent or as center of imagination or thought), where this integration remains anchored in an embodied self. This embodied self is a flexible and variable entity, which we can account for only with a pattern theory of the self (in line with Gallagher, 2013). Furthermore, I outline how this pattern theory of the self fits into the predictive coding framework, which also answers the open question whether self-representation is prior to world-representation or the other way around. The principal organization of a mechanism of building up a self-model is such that both types of representations are always activated and developed in parallel. Modeling oneself is a process which is always activated when one interacts with the world – much as a shadow is present when a person walks in the sun.

## Naturalistic Theories of the Self: Conceptual Clarifications and the Debate About the Self

What is the self and how can we best characterize the phenomenon of human self-consciousness? Core phenomena of self-consciousness include the perspectivity of our experiences, the sense of ownership of body parts (‘this is my arm’), the sense of agency (‘this is my action’), the sense of authorship of thoughts (‘this is my thought’) and the transtemporal unification of a plurality of self-related information into an autobiography (Synofzik et al., 2008b). As a starting point for a systematic characterization, the self can be described as the bearer of self-conscious states. From a naturalistic point of view, the self is a cognitive system that enjoys some form of self-consciousness. Self-consciousness can be defined as the ability to represent one’s own states *as one’s own*, especially (but not only) mental states, where this self-representation is combined with a conscious experience (Newen and Vogeley, 2003). In the case of competent speakers, this involves an indexical representation typically expressed by the word “I”. Yet even where the relevant representation of my own states involves neither language competence nor consciousness, we still have to presuppose a characteristic *immediate self-representation*.^1^ The phenomena to be investigated in principle include explicit self-consciousness, implicit self-awareness and immediate self-representations not accompanied by any conscious experience. These systematic characterizations allow us in principle to ask whether different systems, like animals and robots, enjoy explicit self-consciousness, implicit self-awareness or have self-representations. Focusing in this article on humans, the central question is how to naturalize self-consciousness, be it explicit (easily consciously accessible) or implicit (partially or not consciously accessible). The paradigmatic cases here are those involving immediate self-representations combined with some conscious experiences. The specific aim is to clarify whether a naturalistic theory of self-consciousness needs to presuppose a self at all, or whether the implementation basis of self-conscious states are just processes: the no-self view seeks to explain all the phenomena of self-consciousness by relying only on the brain and its processes without presupposing any entity worthy of being called a self. A self is supposed to be superfluous. I argue that this does not work. In addition, we need to presuppose an embodied self as the basis of all cases of everyday implicit self-awareness or explicit self-consciousness. Even in special cases like ‘out-of-body-experiences’ there remains at least a weak embodied self, even though it may be incorrectly experienced in several respects, e.g., concerning the spatial location of a touch experience.

Why do we need a discussion of the embodied self? Isn’t it by now clear enough that we need to presuppose a self? There has been quite a lot of work on the role of bodily states as regards the ontogenetic development of the self (Bermúdez, 1998; Neisser, 1998; Newen and Vogeley, 2003; Gallagher, 2005). But it does not follow from the observation that bodily self-representations play a crucial role in ontogenetic development that this remains so in all cases of adults being in a state of implicit self-awareness or explicit self-consciousness. We need additional systematic arguments to establish this, and indeed there are influential authors who explicitly argue against the existence of a self (Metzinger, 2003, 2009, 2011; Gazzaniga, 2011). Thus we need a clarification of the role of an embodied self in experiencing self-awareness and self-consciousness. I focus on a criticism of the work of Metzinger, who marshals the strongest evidence and has worked out the strongest arguments in favor of the no-self position. Reviewing many phenomena including mental disorders, Metzinger (2003) seeks to show that body ownership or the sense of agency as aspects of the self are constructs of the brain and can rather easily be modified: e.g., in the rubber hand illusion experiment the rubber hand becomes part of the body schema of a person. He predicted the existence of an analogous full-body illusion, which was then demonstrated in a collaborative study (Lenggenhager et al., 2007). The observation of constructive brain processes which implement the body schema and the sense of agency is a main reason for Metzinger to deny the existence of a self in all respects: “However, there seems to be no empirical evidence and no truly convincing conceptual argument that supports the actual existence of ‘a’ self.” The no-self alternative “could be simply the default assumption for all rational approaches to self-consciousness and subjectivity” (Metzinger, 2011, p. 279). He claims that the phenomena of self-consciousness can be fully explained by presupposing only the brain and its ability to produce self-models: i.e., self-consciousness is explained as relying on processes alone.

Against the idea of the self being any kind of entity, Metzinger argues that the self cannot be a substance in any of the three clear metaphysical senses of the term: (1) The self cannot be a spatio-temporal object because “selves are not to be taken as bodies or biological organisms *simpliciter*” (p. 281). (2) The self should not be identified with a special property of a primitive ‘thisness’ called *haecceitas* since the special phenomenological property of self-consciousness does not imply any special metaphysical structure. (3) The self should not be understood as a unity of features, where this idea of a feature bundle of the self is anchored in Hume’s bundle theory of the self. The reason for denying the third option is simply that it is more parsimonious not to presuppose any self in such a case, although Metzinger explicitly accepts that the classical challenge for how a multitude of features could be integrated into a self can easily be answered in modern cognitive science, e.g. dynamical self-organization in a biological system which realizes a unification of multiple features.

I agree that each of the three classical positions on the self are unacceptable. Nevertheless, the third line of consideration opens the door for a modern re-interpretation and thus a new theory of the embodied self. More precisely: *self-consciousness results from an integration of an embodied, basic affective flow with an intentional object (the self as agent or as center of perception, imagination or thought) where this integration remains anchored in an embodied self.* By the ‘basic affective flow in a situation’ I mean the integration of all activated features in a situation which involve a registration of my own bodily or affective processes, where this can involve the registration of homeostatic features (like body temperature and breathing), sensorimotor features and affective features, as well as of bodily or affective dispositions or expressions. Some of these registrations are typically unconscious (like homeostatic features), and I account for the case that the integration of all these features can be realized without conscious experiences being involved, e.g., if immediate self-representations could be realized in a robot.^2^ Focusing on human self-consciousness, this integration of self-directed features normally involves some conscious experience and thus is a basic affective flow, i.e., a basic self-directed bodily or affective experience. This could be, e.g., feeling oneself as lying in the sun, as being touched on the left hand, as being nervous, as walking through the street, etc. In standard cases, the embodied self is determined by a basic affective flow and is additionally engaged in integrating image-based features as well as cognitive-descriptive features (which both go beyond the affective flow). It will be shown that image-based and descriptive features can be radically incorrect concerning the actual embodied self, and in addition that they may be lacking. In contrast, the basic affective flow which consists in a minimal integration of sensorimotor or affective features is always involved in an episode of self-consciousness, although this integration can also involve incorrect feature representations and its role can be radically diminished in non-standard situations, as we will see.

I aim to expound this view, and show that it is conceptually fruitful and empirically adequate. To do this, in section 2 I clarify the thesis and offer some initial arguments for the existence of the embodied self. In the third section, I introduce a new account of the self which I call the ‘pattern theory of the self.’ This account is needed in order to account for the large variety and flexibility of mental phenomena belonging to self-conscious states. In the fourth section I argue that the pattern theory of the self is supported by processing evidence, and that it can be nicely integrated into the predictive coding framework.

## The Central Thesis of the Embodied Self and Main Arguments for it

### The Central Thesis

A biological or living system is an autopoietic entity which is constituted by dynamical self-organization and self-production (Maturana and Varela, 1991). The biological principles of autopoiesis are sufficient to determine a living being possessing a relatively stable border with the environment, where the living system is dependent on systematic exchange with the environment, for example for nutrition or to eject waste. Such a biological unit is at the same time the evolutionary basis of a cognitive system (Thompson, 2007). If the biological system develops into a cognitive system which is able to have self-representations and to combine them with conscious experiences, then such a system is a self-conscious biological system, as we humans are. The basis for a self is a biological system that perceives and acts in the world, and such a biological system is a self only if it develops self-representations about itself ‘as itself’, i.e., in an immediate way, not representing itself as an object but as the subject of perception and action or thinking.^3^ If such self-representations are integrated in a conscious experience, then this biological system is a conscious self, and if the system is able to integrate several self-representations at one timepoint as well as over different timepoints, then this results in a consciously experienced, multi-feature-involving and transtemporal self. This, then, is a typical human self.

Now we start to explicate the ontological framework in more detail, which includes the existence of a self as based on the biological being with the capacity for self-representations. We need to distinguish three aspects of the self in the new theory: first, the self as the biological being; second, a specific self-relation, namely the ability to form self-representations of oneself in a self-directed manner (i.e., in an immediate, subject-relating way, see footnote 1); and third, the self-model, i.e., the unity of multiple sources of self-information which is integrated by the biological system. In contrast to Metzinger, I argue that we need the embodied self in addition to the self-model and the brain to adequately explain the phenomena of self-consciousness as well as many closely related phenomena.

### Main Arguments for the Embodied Self

Many philosophers who write about the self consider self-consciousness only as realized in a situation, and do not consider that we have to account for long-term features of a self – yet it is the self which may be in a depressive mood for a day, or which may generally have an extrovert temperament. One strategy to prevent the ascription of long-term dispositions to a self is to claim that these should be attributed to a person which is different from the self. But this is a problematic move: the person cannot plausibly be separated from the self without a widening of the ontology. A more parsimonious alternative is to identify self and person at the ontological level, while allowing a distinction, comprising two different aspects or modes, between the notion of ‘self’ with which we usually highlight the epistemic and cognitive features of self-consciousness the subject has, and the notion of ‘person’ with which we usually highlight the practical dimension of its moral rights and duties.^4^ If we accept a self as a transtemporal entity which is ontologically identical with the person, yet characterize different aspects of that entity with the two terms, we can attribute both short-term and long-term mental properties to the same entity, i.e., the self. Furthermore, this position is shored up by a central argument from the philosophy of language: if an English speaker utters “I am hungry” then she expresses the thought that the embodied self (i.e., she herself as a person) is hungry. Yet Metzinger, by denying the self, has to reconstruct the utterance “I am hungry” differently: not as an utterance about an embodied self but only about the self-model which contains bodily representations. I accept that the content of the utterance “I am hungry” is part of the self-model when the thought expressed is realized, but “I” does not thereby refer to the self-model because this would imply that the content of the utterance is that the self-model is hungry – and this is absurd, since contents cannot be hungry, sad or happy. The self-model as a unity of self-relating information is produced by the brain but anchored in the embodied self, i.e., the self-representing biological system which realizes this self-model. As a consequence of this position, the reference of “I” is the biological system and not any content, and a fortiori also not the self-model. This is exactly what the philosophy of language has come to agreement on concerning indexicals (Kaplan, 1989; Perry, 2001) and proper names (Kripke, 1980; Künne et al., 1997). Their reference is an object and this object is also the contribution to the truth-condition which is determined by the utterance of the relevant sentence containing the expression. The position that I am an embodied self thus fits nicely into modern philosophy of language, while Metzinger’s no-self account is in conflict with it.

Metzinger’s main line of interpretation for all self-consciousness is that the self is a fiction. There is only a phenomenology of a sustainable self produced by the content of the self-model, alongside the fact that we have a “transparent” interpretation of the contents of the self-model: we experience them as referring to something, being about something, even though they do not refer at all (Metzinger, 2011). Metzinger has to presuppose that we are systematically and completely mistaken by presupposing an embodied self. But this is evolutionarily implausible since the realist view about ourselves as embodied entities is critical to enable us to act now and plan out actions adequately. Furthermore, there is evidence that our basic self-representation is energetically quite costly for the body, since it is connected with the so-called resting state of the brain which is also massively overlapping with the cortical midline structures (Vogeley and Fink, 2003; Northoff and Bermpohl, 2004; Qin and Northoff, 2011): independent from any details, a large part of the brain is permanently active in producing self-representations. From an evolutionary point of view, it would be difficult to explain this intense energetic investment of brain processes if the basic self-representation were not pragmatically successful. And, in addition – the step Metzinger would deny – the latter is often combined with a roughly adequate view of the world, e.g., as Newtonian mechanics gives us a roughly correct world view which still holds according to Einstein’s theory, although only for standard conditions. In the same way, the arguments support the view that the embodied self is an adequate view of ourselves in standard conditions although it may need some qualification for special situations; whereas Metzinger argues that our view of an embodied self is radically wrong. Let me introduce a comparison in order to strengthen the claim that the energetically costly view of ourselves as embodied can be taken at face value. The underlying mistake of Metzinger’s claim can best be analyzed by drawing a parallel between the self and an everyday entity like a table. The self is in many respects – contra Metzinger – a real entity, to the same degree as a table is.

One way to reconstruct his underlying no-self argument (according to Metzinger, 2003, 2009) runs as follows:

(1) Experiencing the self is a construct of the brain. Constructs of the brain are fictional contents. Thus there is no self, but only a brain and the fictional content of the self-model constructed by the brain.

The problem with this argument is that it also proves that this table I am sitting at does not exist:

(2) Experiencing this table is a construct of the brain. Constructs of the brain are fictional contents. Thus there is no table, but only a brain and the fictional content of a table constructed by the brain.

Accepting this argument would lead to a radical anti-realist position, since a presupposition of this debate is that we are searching for a naturalistic ontology^5^ and we accept either a naturalistic realism or a naturalistic constructivism. Given the discovery of the rubber hand illusion and out-of-body experiences as systematic constructions of the brain (Lenggenhager et al., 2007), Metzinger argues that the self is not real but only a constructed fictional content, whereas he accepts a normal table as being a real object. But given that my experience of the table is a construct of the brain – which is commonly accepted in perception science –, it does not follow that the content is a fiction, i.e., that there is no table, quite the contrary: given the evolutionary pressure on our visual system, there is almost always a table present when my brain constructs a visual experience of a table (with the exception of special visual conditions). Generalizing, we can presuppose a minimal realism for everyday objects like tables, cars and human beings, which for independent reasons most philosophers, including myself, think we need to accept. Then the argument from brain constructivism to anti-realism above has no bite. Being a constructed content of the brain does not imply that there is no instantiation that corresponds to the content. The self can plausibly have the same reality as the table I am sitting at, and this is sufficient for it to be real even though it leaves open whether the ultimate ontology for tables is one in which they should be reduced to atoms, electrons, and other particles and their physical properties. If one has a fundamental metaphysics according to which tables do not exist, the claim that selves do not exist lacks any special bite, because we have then changed the game from an interesting ontological debate about specific entities (according to which tables exist, but not a self) to a general debate between minimal realism on the one hand, and radical constructivism of all sensory experiences, on the other.

What is further positive evidence supporting the embodied self as a basis for unifying further self-related contents into a self-model? We have knowledge-how concerning how far we can reach, i.e., about our peripersonal space, how high we can jump, whether we can pass through a narrow gap given our own particular height and width, etc. Such embodied dispositions are described as constituting an implicit body schema. This is a challenge for the self-model theory if – as argued in Gallagher, 2005 – this implicit body schema needs as its realization basis not only sensorimotor brain activation (which could be evaluated as part of the neural basis of the self-model) but in addition the actual body limbs and their specific connection with the brain. Features like the feeling how fast I can run can become manifest in a situation, but they are always present as a dispositional part realized in the embodied self which is going beyond the self-model. Furthermore, a self includes its affective and emotional life: emotions are essentially embodied and this includes affective dispositions like moods or even long-term emotional temperament. After half a century of debate between feeling theories and cognitive theories of emotions, no purely cognitive theory of emotions has survived criticism. Thus, the only remaining candidates are variants of feeling theories (LeDoux, 1996; Prinz, 2004; Barlassina and Newen, 2014) or mixed theories of emotions (Scherer, 2009; Newen et al., 2015). Since emotions essentially involve an embodied affective flow, and an affective, emotional life is a constitutive part of the self, this gives us another source of evidence for the embodied self. So far, then, we have many reasons to accept a self, and have defeated one of the main arguments of the no-self position. Thus, we already seem better off accepting an embodied self. But what exactly is this embodied self?

There are many convincing arguments that the self is not a substance (Metzinger, 2011) in the metaphysical sense of an entity which has constitutive features which are necessary (involved in all possible worlds) and jointly sufficient: the self lacks clearly specifiable identity criteria in this classical sense because it has so many facets.

We have to distinguish the self as subject from the self as object (e.g., Brockner and Wiesenfeld, 2016). The self as subject involves the self as (1a) bearer of one’s sensations and perceptions, (1b) as the agent of one’s action, (1c) as the owner of one’s body parts, (1d) as the center of one’s visual perspective and (1e) as the center of one’s cognitive perspective (including experiencing oneself as the author of one’s thoughts) (Synofzik et al., 2008b). Furthermore, *the self as subject* is characterized by its immunity to error through misidentification (Prosser and Recanati, 2012), i.e., it involves immediately self-related information such that the system the information is about cannot be misidentified because there is no identification involved, but just a specific kind of information processing that makes it about the system, where proprioception is such a type of information: the self as subject always contains information about itself even if in rare cases the proprioceptive information is evaluated incorrectly, e.g., one may think one’s legs are crossed when they actually are not. *The self as object* involves recognition and conceptualization of the self, which is e.g., manifest in mirror self-recognition, which infants usually learn by 18 months. Another manifestation is any form of explicit conceptualization and description of oneself. The self as object always involves an identification process. Explicit contents of oneself constitute the so-called narrative self. In our everyday self-experience, these theoretical distinctions between self-as-subject and self-as-object are not manifest since the phenomenal self-experience in a situation is based on the integration of all self-relating features that are activated in this situation. Given such a plurality of features which can manifest in a self, does it still make sense to presuppose the self as an embodied entity, or are we better off accepting that the self is only a “useful, heuristic posit” as Metzinger claims (Metzinger, 2011, p. 280)?

## The New Account of the Embodied Self: the Pattern Theory of Self

To make a convincing case for the embodied self as being a real entity, I develop what I call the pattern theory of the self. This allows us to account for the variety and flexibility of self-conscious phenomena. The self is the embodied human being, while the self-model is an integrative pattern of characteristic features which is anchored in the body and which determines the body as the anchoring unit for self-conscious experiences. The integrative pattern of characteristic features unifies all kinds of self-directed information (a detailed application of the pattern theory is given for emotions in Newen et al., 2015). This idea of a pattern theory of the self involves a new understanding of what it means for the features of the self to *be constitutive*; this needs to be distinguished from the classical metaphysical understanding. The latter says that a feature X is constitutive for a phenomenon of type P if and only if it is necessarily (in all possible worlds) involved in all realizations of P. Such a strict understanding is used in standard metaphysics and may be helpful within a fundamentalist ontology which aims to investigate the fundamental physical particles which are the building blocks of everything. But such a perspective is completely misguided for a minimalist ontology in the philosophy of mind. Mental phenomena classified by folk-psychological terms are of course realized by brain processes, but they never satisfy such a strict criterion. Even in the case of culturally universal basic emotions (according to Ekman, 1972) like fear, we do not find a clear group of necessary features including brain processes realizing them in the strict sense (Barrett, 2006; Newen et al., 2015); a fortiori, it is plausible that we will not find necessary features for more complex mental phenomena. Thus, for a philosophy of mind and cognition we need a more modest notion of *being constitutive*. But such a notion still has to respect the insights concerning the empirical foundations of the relevant phenomena. The central claim is that *the self is individuated as an integrated pattern of characteristic features*, where all the features are constitutive for the self as a type without all of them being involved in each token of the self. On the contrary, there is a great variety of integration of features, and the features involved can vary quite intensely. This calls for a new explication of what it means to be constitutive.

Here is a new suggestion that I have already used for the discussion of emotions, which are also shown to be individuated as patterns of characteristic features (Newen et al., 2015): the core idea is that even a very few characteristic features can be sufficient for an emotional episode to be a token of a certain type, e.g., *fear*. Those causal features that contribute to an emotional episode of fear are considered constitutive if they are not dispensable for the episode without losing the status of being a token of fear. On an interventionist notion of causality, if one intervenes by subtracting a particular causal factor which makes the fear dissipate, then that causal factor would be considered constitutive and part of the pattern that makes it an instance of fear. More technically, I suggest the following notion of being constitutive for a phenomenon X given that X is organized as an integrated pattern of characteristic features: a feature F is constitutive for a type of pattern X if it is part of at least one token set of features x which is minimally sufficient to belong to the type X. ‘Minimally sufficient’ means that these features are jointly sufficient for the (token) episode x to be of type X, but if one of them were taken away the resulting episode x′ would no longer count as an instance of X. It might then be a similar but different phenomenon, e.g., if an emotional episode involves a typical facial expression of fear and a cognitive evaluation of the situation as dangerous and a certain level of arousal, but no intense negative affect, it may no longer be a token of fear but rather of being worried. This understanding supports our view of the pattern of characteristic features described for fear above. This account opens a middle ground between metaphysical necessity in the classic conception of constitution, and the overly liberal view that allows any causally relevant feature of a phenomenon to be constitutive for it (thereby denying any difference between being causally relevant and being constitutive). This new middle ground is suitable for phenomena that are organized as patterns, and I now argue that the self is one of those phenomena.

Let us first remind ourselves of what the self is not: the self cannot be characterized as a rigid entity which never changes any of its constitutive features: this would prevent us from accounting for the variety of phenomena of self-consciousness – yet this does not imply that it is not real. We turn, then, to what the self is: the self is a flexible entity which is a unity of characteristic features integrated as a pattern in a situation and then developed further. The features and its integration constitute an embodied self including a short-term self-model in a specific situation, as well as a long-term self-model which may slowly modify its features such that we can understand how a 3-year-old child can transform into a young lady while retaining a transformed but continuous self. The self as an integrated pattern of characteristic features has many aspects that parallel the reality of tables: tables are natural entities which on the basis of physical properties also have supervening relational properties like being flat, being handy for putting stuff on, etc. A self is a natural entity which on the basis of neural processes also has supervening relational properties, like being an agent in the environment, and affordances like having a certain range in reaching for objects, etc. Can we systematize the characteristic features which are candidates for constituting a self in a situation? Yes, we can.^6^

The self is a biological system – here a human being – which has the capacity for self-consciousness, where the latter is realized in immediate self-representations which are consciously experienced and integrated into a pattern. Such a pattern normally consists of (1) typical affective and vegetative features like homeostatic processes and a body-centered frame of reference which are the foundation for an evolutionarily basic distinction between a biological system and its environment; (2) typical behavior or behavioral dispositions like self-directed dispositions of self-care for bodily conditions and for psychological conditions (e.g., via self-deception) and for social conditions such as being a self in different groups; (3) self-directed expressive behavior like self-directed gestures, body postures and speech; (4) experienced features such as a first-person perspective, a sense of ownership of body parts, and a sense of agency over one’s actions; and (5) cognitive features such as explicitly imagining oneself or thinking about oneself and telling autobiographical stories about oneself. Furthermore, each episode of self-consciousness has (6) an intentional object, namely the self, i.e., the self-representing biological system, respectively, the human being.

For each episode of self-awareness or self-consciousness we need an integration of a minimal package of features into a pattern such that the result of this integration is the self as an intentional object. Thus, an episode of self-awareness or self-consciousness is directed toward oneself as an embodied entity.^7^ It is important to note that the cognitive contents and the experienced features can be misguided, especially in cases of mental disorders: the sense of agency is misguided in the case of ‘alien hand syndrome,’ where someone experiences his own hand as belonging to someone else. Another example is the ‘anarchic hand,’ where someone experiences the hand as one’s own but as being out of control, i.e., as doing what it wants, not what the owner intends. The sense of agency is distorted while the sense of ownership can be adequate. Nevertheless, in all these cases there remains a core embodied self as the intentional object even though some features about the object are distorted. This view allows us also to account for thought insertion as a symptom of schizophrenia: many aspects of the affective flow remain adequately integrated and thus there is a normal bodily self-awareness which is combined with an adequate sense of ownership of body parts; in addition, there is some irregular processing leading to strange experiences together with a local breakdown in the rationalization strategy (Vosgerau and Newen, 2007) which results in an attribution of authorship of one’s own thought to someone else. The pattern theory of self enables us to account for many mental disorders of self-awareness in the following way: as long as there is a minimal anchoring of our awareness in the embodied self, i.e., in the physical body which seems to be guaranteed in (almost) all situations (see section “Discussion” below), the actual embodied self is the intentional object of the awareness even if substantial parts of the content of the self-model are strangely experienced or misattributed. Further challenges like ‘out-of-body experiences’ will be discussed later.

### Arguments for the Pattern Theory of Self

The main claim we have to defend by reference to empirical cases is that features can be constitutive for the self in the new sense even while those features are not thereby necessarily involved in every form of self-consciousness. The most obvious case is that we can of course have an episode of self-consciousness while not making any *self-directed expressions*, especially if we are trained to avoid gestures, overt body postures or speech in relevant situations. Nevertheless, such self-directed expressions like making an utterance of an “I”-sentence, sometimes stressed by a pointing gesture to oneself, are very typical. But why should we evaluate these expressive features as constitutive for being in a state of self-consciousness? This is plausible for any view of the mind in which the state of the mind is not completely independent from the expressive behavior. One need not hold a radical expressivism according to which internal mental phenomena are reduced to their expressions. It is sufficient to accept that sometimes the expressive behavior is part of the mental phenomenon and not merely a report or commentary about it. Arguments for a minimal role of expressive behavior in the case of self-knowledge have been proposed by Bar-On (2004). They are similar to the arguments supporting that a facial expression of fear is part of the basic emotion FEAR (Newen et al., 2015): this is convincing since there is a typical facial expression of FEAR and this expression as well as its recognition is culturally universal (Ekman, 1972). Thus, it is plausible that the expression is part of the emotion FEAR even though we can train not to show it (making a poker face).

When are cognitive features lacking? A consideration of the phenomenon of the sense of agency, for example, suggests that we can have a sense of agency without any specific cognitive evaluations. To make this clear, we have to distinguish between the *feeling* of agency, and the *judgment* of agency which can be independently implemented (Synofzik et al., 2008a). E.g., when I am driving my car in an automatic but controlled manner along my normal route to work, then I may have a feeling of agency without any judgment of agency, especially if my mind is completely taken up with other thoughts. In the other direction, if I am alone in a small kitchen and suddenly an object falls down onto the floor, and I do not have a feeling of having touched it (although in fact I did), I do not have a feeling of agency concerning the falling of the object, even though the best explanation is that it must have been me. Thus, I may immediately develop a judgment of agency about having done this even while the feeling of agency is lacking. The same distinction has to be made with the sense of ownership of body parts, i.e., to distinguish the feeling of ownership and the judgment of ownership (Synofzik et al., 2008a,b).

**FIGURE 1:**
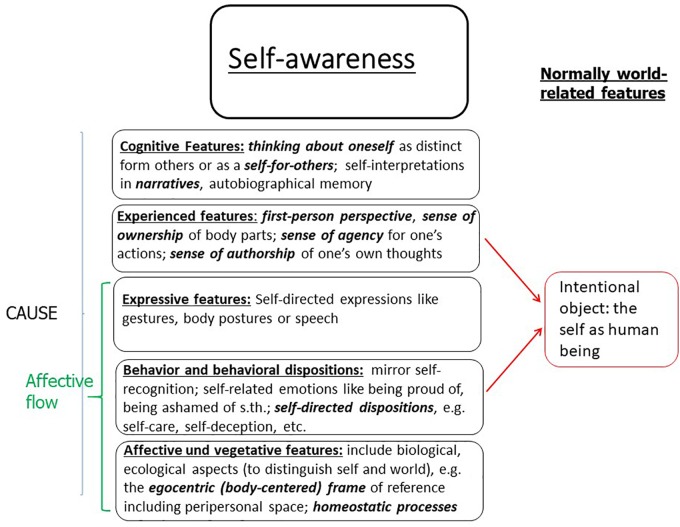
The framework of a pattern of self-awareness (or self-consciousness).

The basis for one of the typical self-conscious states like the sense of agency, ownership or perspectivity, is some minimal integration of self-directed information which, in the case where a conscious experience is involved, is affective flow. Since our focus is on paradigmatic cases involving a conscious experience, the basis of self-consciousness is normally an affective flow which can be characterized as a basic self-directed bodily or affective experience. It can have different sources (see Figure 1), e.g., either certain self-directed affective and vegetative features, or certain self-directed behavioral dispositions or self-directed expressive behavior or a combination of these features. A minimal combination of these features can establish a basic self–world distinction. Thus, we can have quite a variety of implementations of a basic affective flow. But can such a self-directed feeling be lacking completely in a conscious human being? And would this not deliver a counterexample to the thesis that an embodied self is the basis of self-consciousness? Such an example is only available in a case of severe mental disorder, e.g., the Cotard syndrome. A Cotard patient experiences himself as not having a living body, as being dead. An explanation is that the patient either does not have any affective flow, or at best a radically diminished one, even though the cognitive ability to form self-conscious thoughts is still intact. The person can act in the world, but has no motivation to do so. Thus, the lack of affective flow produces the situation of an almost pure cognitive self-reference expressed by “I am dead,” while the affective flow and the conscious experience of agency, ownership and perspectivity is radically diminished. Is this a counterexample against the embodied self as the basis of self-consciousness? No, it is not, since the pattern theory can best account for the large variety of phenomena including Cotard syndrome: the registration of an affective flow is not completely missing since the person still registers some attachment to the body, although it is so weak that it is evaluated as one’s own body experienced as being dead, due to a lack of bodily warmth or emotional arousal. This interpretation fits with the neural investigation of a particular case of a Cotard patient (Charland-Verville et al., 2013): the PET study shows a highly reduced activity of the precuneus (but still some remaining activity) which is a central component of the network responsible for introspective experience, i.e., registering one’s own bodily and affective states. Thus, the Cotard patient has a kind of almost pure cognitive self-consciousness, and remains connected to their own body although the strongly diminished affective flow leads to a radical misinterpretation of the properties of the embodied self. Since the patient can walk and act while feeling dead, we can characterize this special situation as ‘phenomenological disembodiment’ which exists alongside quite elaborate forms of functional embodiment.^8^

To sum up this part: self-consciousness is the result of an integration of a basic affective flow and experienced features with an intentional object constituting an embodied self, where the integration typically also includes cognitive features. We have seen that cognitive features can be lacking (e.g., in the case of a feeling of agency) or that the basic affective flow and further experienced features can be radically diminished (e.g., in the case of Cotard Syndrome). The pattern theory allows for and predicts a variety of phenomena of self-consciousness which remain connected with an embodied self, although in extreme cases rather loosely. Furthermore, the properties experienced or cognitively self-attributed to the embodied self could be incorrect as a result of the integration process. Thus, we cannot suppose a self to be a stable substance as classical metaphysics suggests, but equally we do not have to retreat to a no-self thesis. The self can best be described as a flexible embodied self which is the result of an integration of typical features into a minimal pattern. And such a self cannot only account for the integration in a particular situation, but also for a long-term integration expressed in dispositions and autobiography. This is the place to fully account for the thesis of the ‘narrative self,’ according to which the self is the unity of stories someone tells about himself or herself (Dennett, 1992; Schechtman, 2007; Hardcastle, 2008): the ‘narrative self’ can be analyzed as the descriptive content of the embodied self. Thus, the self is not abstract but real and embodied, while the embodied self can be partially characterized by long-term autobiographical contents and those are usually taken to be rather telling for human beings. Taken together, the embodied self is best understood as an integrated pattern of characteristic features, where this allows for wide flexibility in the relevant features.

### Further Advantages and Fruitful Applications

There are further advantages of accepting an embodied self as the normal standard. For one thing, we can better account for the wide variety of data from bodily illusions such as the so-called rubber-hand illusion: the illusion that a rubber hand is one’s own hand can be produced if my actual hand and the rubber hand are synchronously touched, where I feel the touch on my hand but perceive it on the rubber hand. Thus, the rubber hand illusion is based on an affective flow of the body which is synchronous with the perceptual experience. A simple request that one should imagine the rubber hand as being one’s own does not produce the illusion. This can also be demonstrated by indirect effects: if someone has the rubber hand illusion and another agent starts to bring a hammer down onto the hand, the person automatically pulls back the hand (although she cannot control the rubber hand), while in the case of mere imagination there is no such reaction. An interesting variant is the so-called “cardiac rubber hand illusion” (Suzuki et al., 2013). There a rubber hand is caused to flush in the actual rhythm of one’s heartbeat. The heartbeat which is synchronous with the perceived flushing of the rubber hand is sufficient as an affective flow, and thus the illusion is triggered. The illusion is anchored in the embodied self which has an affective flow, and on the basis of this together with perceptual experiences can reintegrate its features into a modified self. An affective flow of the embodied self remains the anchor of rubber hand illusions or full-body illusions, even though in such cases the integration process leads to pragmatically incorrect experiences like out-of-body experiences (Lenggenhager et al., 2007).

**FIGURE 2:**
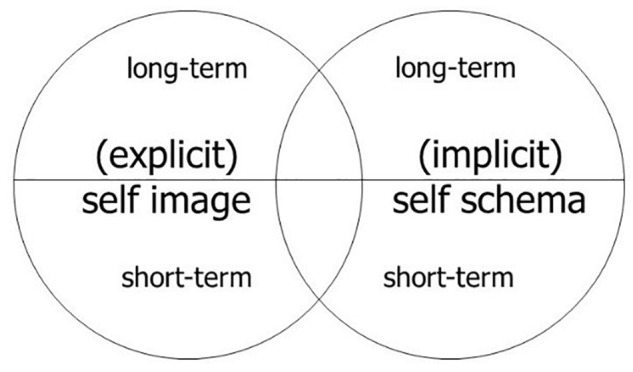
The self-model and its structure.

**FIGURE 3:**
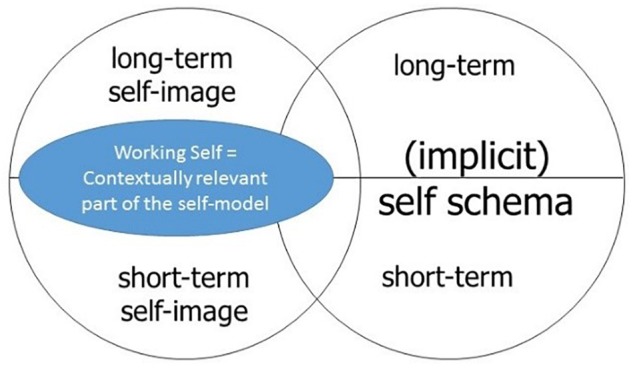
The self-model and the working self.

Another advantage of a pattern theory of self is that it offers a way to address the challenging question about the relation between self and memory. The pattern theory of self allows us to account for the working self-model based on working memory and for a long-term self-model based on long-term memory, while the embodied self is the result of integrating all the activated self-relational information in a particular situation or for a longer period. The long-term features are not considered in Metzinger’s work, since he investigates self-consciousness only as a process in a certain situation and thus cannot adequately account for embodied long-term dispositions which are clearly part of the self, e.g., being extroverted, being stingy etc. Such things are mainly contributed by procedural memory, and this contribution can be easily accounted for within the pattern theory since it is just another contribution which enters into the integration of an embodied self, which also involves experiential and cognitive contents. To make the difference from Metzinger and any no-self position clearer, we can use the terminology already introduced, namely the self as a biological system with the ability of self-consciousness and the self-model as the unity of contents of all self-related information. The pattern theory of the self involves two claims by way of accounting for long-term dispositional features of the self, namely, first, *that in addition to the self-model, we have to accept that there is an embodied self*:^9^ as already indicated, we are definitely in need of the latter, e.g., to account for long-term dispositions in contrast to the situational features of the self. The second claim concerns the inclusion of these long-term dispositions which are anchored in procedural memory and are often not easily accessible or at least not accessed in a situation even though they still guide the behavior of the embodied self, e.g., the disposition of being extroverted guides the behavior of a self in all situations, including in situations in which one is not aware of this feature. Thus, *it is helpful to distinguish the implicit self-model from the explicit self-model, where the latter includes all the information that is easily accessible^10^ and the former includes only information which is rather difficult to access or never accessible* but still relevant for the embodied self and its behavior or behavioral dispositions. Focusing on the self-model only, the two independent distinctions (working self versus long-term self and implicit versus explicit self-model) allow us to draw a more adequate picture of the self-related information and its relevance for the embodied self: a self as a cognitive and behavioral system with the ability of self-consciousness has a very rich package of self-related information included into its self-model. All this information can be split up into short-term versus long-term information, as well as explicitly available information integrated in the self-image versus implicit information integrated in the self-schema. Metzinger focused mainly on that self-related information which is consciously accessible and activated for the short-term working memory, i.e., the lower half of the left circle (see Figure 2). A more adequate picture would be that such a cognitive and behavioral system has a rich self-model which is the union of the explicit self-image and the implicit self-schema, although in certain situations only a contextually relevant part of the self-model is activated (see Figure 3). But the working self must still involve explicit as well as implicit information if we want to describe the embodied self adequately concerning all its behavior, its behavioral dispositions, and its explicit autobiography (as expressed in narratives in a situation).

The self-model receives its information from the memory systems which include short-term memory (working memory) and long-term memory, while according to standard accounts the latter is divided into declarative memory (semantic memory as well as episodic memory) as well as non-declarative memory (procedural as well as associative memory). The different memory systems are the sources of the self-model which is the content of the embodied self. This is one core aspect of a highly interactive Self-Memory System (Conway, 2005). This is the place to integrate the so-called narrative self: it can be characterized as the contextually relevant part of the explicit self-model, where this includes all the self-descriptions in a situation. A detailed description of the interdependence of self-model and memory systems would go beyond the scope of this article, which is limited to describing, first, the role of the embodied self, and second, the limitations of the no-self position. With the relation to the memory systems as important sources especially for long-term aspects of the embodied self, we can furthermore argue (i) that the self-model is systematically connected to and anchored in the embodied self even in inadequate imaginations, and (ii) that we need to presuppose an embodied self even when all cognitive self-evaluations are lacking.

Ad (i): The first part can be made explicit by asking whether my own perceptual images of myself can completely disconnect me from my bodily experiences. In an experimental setting we can develop an out-of-body experience as an interesting candidate for a non-standard perceptual image: we are stroked on our backs by the experimenter, and our back is filmed. The movie is simultaneously sent to computer goggles which allow me to perceive this stroking of my back, although I perceive myself as being three meters in front of me. This out-of-body experience is a full-body illusion employing the same mechanism as the rubber-hand illusion (Lenggenhager et al., 2007). It is presupposing an affective flow realized by feeling the stroke on my back, and the melding of touch and perception leads to a shift of the location of the experience toward the perceived virtual body. Since the affective flow is involved, there is a bodily feeling which remains constitutive for my self-experience even if I mislocate the experience. Further evidence for the central role of embodiment comes from a recent experiment about dislocating myself into a non-human-looking humanoid robot body which I steer while wearing computer goggles, thereby seeing through the eyes of the robot. The interesting observation in such a setting is that humans are able (at least subjectively) to bi-locate in two different bodies at the same time, the actual and the virtual body (Aymerich-Franch et al., 2016). In the same way we may analyze the self through dreams which remain anchored in the affective flow of the actual body even if in the dream one is flying, although there is a debate over whether this may be a purely temporal consciousness (Windt, 2015, 2018b). But even in these cases the position of weak embodiment is plausible since an affective flow of the actual body is strongly intertwined with our perceptual images.^11^ The latter is the case if we accept that in each of our minimally conscious moments we have an existential feeling (Ratcliffe, 2008) which can best be characterized as produced by non-cognitive low-level homeostatic or bodily processes which produce an existential feeling as a result of being sensitive to one’s blood-flow, heart-beat, physiological pressure of standing, sitting or lying, one’s hand touching one’s head, one’s visceral processes etc. Such a basic self-awareness is in line with the work of Damasio, who calls this dimension of the self the ‘core self’ (Damasio, 1999).

Ad (ii): The second part can be made explicit by asking whether there can still be an embodied self despite a radical decrease of semantic and episodic memory as the sources of the long-term self-model. Concerning a loss of the ability to encode any new episode into episodic memory, there is the well-known case of the patient H.M. Due to medical treatment, the hippocampus was bilaterally removed. H.M. had no marked deficit in the cognitive capability to structure experience in an actual situation, and most importantly he could also learn new abilities. He had no deficit in the ability to form I-thoughts in an actual situation, and semantic and procedural memory remained available. Thus the loss of episodic memory alone, despite its dramatic impact on the subject who henceforth lived his life in 3-min-packages (with every new event then forgotten), does not disrupt an embodied self. What about the radical case of intense Alzheimer’s disease? Fuchs (2018) describes the following case study:

A 78-year-old person suffering from intense dementia most of the time could not recognize his relatives. He was lethargic, solitary, physically weak, and almost unable to walk unaided. One day his grandchildren visited him and played football. At a young age he had played football in a club. Suddenly he stood up and played with the two boys. In contact with the ball he showed them his special skills and tricks and offered expert explanations. For half an hour his dementia seemed to be gone. (Fuchs, 2018; translation from German into English mine).

The case study shows that on the basis of mainly procedural knowledge like the ability to play football it makes good sense to speak of an embodied self: it is the old man who has still the skill associated with some remaining bits of semantic knowledge, but this skill is activated only contextually; thus it seems fruitful to presuppose an embodied self to explain and predict the patient’s behavior. This is supported by observation of the role of procedural memory in dementia and Alzheimer’s patients (Eldrige et al., 2002; Fleischman et al., 2005; Harrison et al., 2007).

Furthermore, we need to presuppose an embodied self as the moral agent who deserves blame or praise, and as the partner of interaction in any social interaction. Neither complex human social communication nor demanding ethical acts can be realized without minimal self-consciousness. Thus, the partner in complex social interactions as well as the moral agent could best be described as an entity with minimal self-consciousness, and according to my position this is the embodied self. Again, the partner in social interaction and the moral agent are not simply brains and their self-models, but embodied human beings with brains and the ability to create a self-model.

A final consideration to support the existence of an embodied self as realized according to the pattern theory of the self would be questions concerning implementation: how can such a flexible integration of the self be realized, what might be the underlying cognitive architecture and mechanism? The outline of the mechanism has two functions: first, it shows that the proposed theory of the self is compatible with a promising cognitive framework, and secondly it allows us to answer the additional question of whether self-representations are prior to world-representations or the other way around.

## Self-Modeling in the Predictive Mind

It is argued that the underlying mechanism can be described by a slight and plausible modification of the highly successful framework of predictive processing (Hohwy, 2013; Clark, 2016). This rests on the assumption that biological systems act, perceive or think efficiently in the world by maintaining themselves in a limited set of expected states. In order to do so, whenever such a system finds itself in a highly unexpected state it must be able to minimize a quantity known as free energy (Friston and Stephan, 2007; Friston, 2010), which is a measure of how much the current state deviates from the expected one, otherwise it will face danger. This idea can be extended to an organism’s cognitive systems by positing a hierarchical internal model of the world that the system uses to generate and revise hypotheses and predictions about its sensory input. The system can test its prior hypotheses by comparing predicted sensory states with the actual sensory inputs. In the case of mismatch, it would be appropriate to revise the model in order to generate more accurate predictions. According to a generalized interpretation of predictive processing (as for example in Friston, 2010; Hohwy, 2013), the job of the brain is to minimize prediction error which is a measure of the mismatch between the expected and actual states of the cognitive system. Starting with prior hypotheses on different levels in a large processing hierarchy, the cognitive system is able to produce an adequate representation of the external world. This has been successfully described for perceptual and motor processing.

Let me now introduce the basic idea concerning the self. We have to presuppose two parallel hierarchies of processing, one based on prior hypotheses for the external world, and one based on prior hypotheses for the self. The self-related information is integrated into the self-model, and in certain situations into the contextually relevant part of the self-model which is the working self. The self is a product of the same sensory information which is used by the cognitive system to construct and modify hypotheses about the external world as well as hypotheses about an internal self.

**FIGURE 4:**
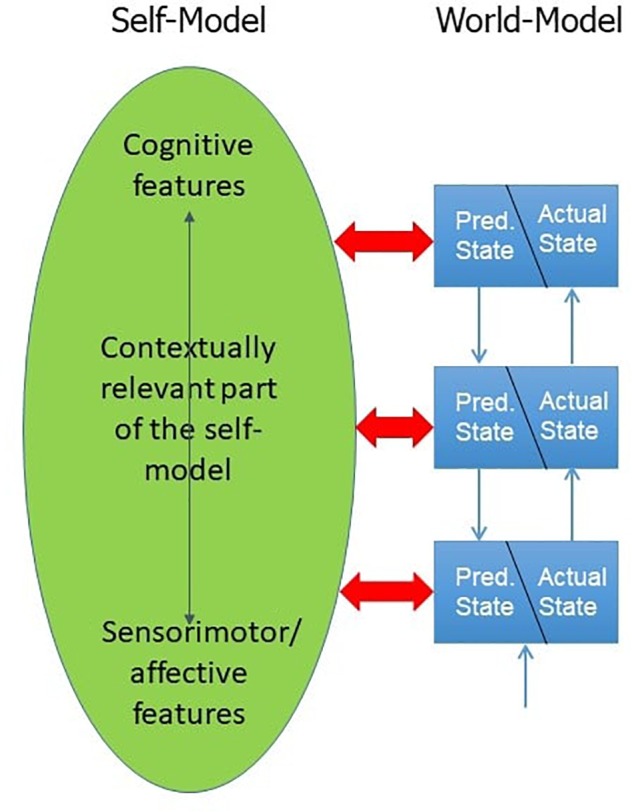
Modeling the self and the world.

To defend the picture, we need to show that the sensory input processed in the hierarchy is at the same time not only producing representations of the external world but also representations of the cognitive system itself (see Figure 4). Why is this plausible? If someone grasps a glass, the neural signal transported into the brain carries information both about the glass and about the hand. The latter is obvious if, e.g., the hand hurts when it touches the glass. But even in normal situations, information is delivered about the grip of the hand, its position, the change of the tension of the muscles in the hand and arm during the grasp, etc.: there is a lot of egocentric information which is processed and integrated at least into the implicit self-model concerning the bodily features [sometimes called the body schema (Gallagher, 2005)]. Not only for such basic cognitive tasks like grasping an object but for all cognitive tasks in which we interact with the world, we receive sensorimotor and affective input and construct information about the world and information about ourselves at the same time. Thus, self-related information is only one side of a single coin whose other side comprises world-related information, and both types of information unfold with the cognitive development during ontogeny (Newen and Vogeley, 2003). Let me offer some more evidence for this claim. There is a conceptual argument concerning the intertwinement of first- versus third-person visual perspective taking. If we distinguish *having* a first-person perspective (1PP) from *being sensitive to* one’s own 1PP, then we may say that the former is already involved in the emergence of the most basic self–world distinction. The sensitivity to one’s visual 1PP comes later; and what could it mean to be aware of one’s own 1PP unless one can distinguish it from the third-person perspective (3PP) of someone else? The same conceptual consideration holds not only for visual but also for cognitive perspective-taking, where the latter means the ability to explicitly distinguish between one’s own beliefs and the beliefs of others. The prediction of this view is that the ability to distinguish 1PP and 3PP involves intense overlapping brain activation, albeit that some specific activations are relevant in accounting for the difference in the perspective: the first study on the difference between the 1PP and 3PP cognitive perspective by attributing beliefs to oneself in contrast to someone else has already confirmed this expectation (Vogeley et al., 2001). In the meantime, a systematic review of the neural correlates of self versus others shows that they have interesting neural overlaps and shared neural activations (Decety and Sommerville, 2003), despite of course there also being differences responsible for the self- or the other-dimension (Vogeley et al., 2001, 2004). It is still an open question what exactly is the role of the shared neural activations for self and other. In the pattern account of the self we expect there to be an overlapping integration process which is then modulated by additional brain resources resulting in either unified self-representations or unified representations of another person. And exactly this is the best interpretation of the data by Lombardo et al. (2010), who have results which “demonstrate that identical neural circuits are implementing processes involved in mentalizing of both self and other and that the nature of such processes may be the integration of low-level embodied processes within higher level inference-based mentalizing.”

Having defended the claim that the same sensorimotor and affective information from the world normally also gives us information about ourselves, the remaining question is how such a process can be realized in a predictive coding framework. To repeat the core idea: any input also involves self-related information and is integrated into the self-model, and in certain situations into the contextually relevant part of the self-model which is the working self. Since the hierarchy of predictive processing starts with quick-changing sensorimotor and affective information and continuously unfolds into slow-changing, high-level cognitive information, the same also holds for the whole hierarchy of self-related information which is integrated into the working self. The general solution I offer consists in outlining how the relevant self-model can be developed and modified by the same external inputs via two parallel integration processes, one about the world and one about the self. This perspective is supported by the work of Limanowski and Blankenburg (2013) who argue that minimal phenomenal self-consciousness can be adequately characterized in the framework of predictive processing. Let us come back to the embodied self. The special role of the embodied self in normal conditions is becoming clear within this pattern account: in normal conditions we receive permanently changing sensorimotor and/or affective information which is also used to construct self-related information, and the latter constitutes an affective flow. Thus, an affective flow of self-related information is normally the basis of the integration of all contextually relevant self-related information into a working self. Thus, the embodied self is the core of our contextually relevant self-model which can only be diminished in rather special conditions, such as Cotard syndrome.

This account has one interesting upshot for the open questions (1) whether self-representations are prior to world-representations, and (2) whether self-understanding is prior to understanding others, as is held by all representatives of simulation theory (e.g., Goldman, 2006), or the other way around (e.g., Carruthers, 2009). Given that the processing of self-related information and world-related information is taken out of the same package of neural signals, the question of priority does not make any sense. A correlate of this is that even if a person is focusing on self-related information, e.g., by registering being the agent of moving a book back onto a shelf, she at the same time activates world-related information, e.g., interacting with the book and the shelf in a specific way. And it works the other way around: even with a focus on world-related information, we gain self-related information at the same time. Thus, in any experience we are constructing both, information which we integrate into a self-model and information we integrate into the world-model. Even if we are focused on modeling the world we still model ourselves, and the other way around: self-modeling is like the shadow of interacting with the world and the other way around; and this holds for conscious as well as unconscious processing of a minimally complex cognitive system. Since humans are furthermore hyper-social beings who right from birth are intensely socially sensitive, we can generalize that there is an intense intertwinement between constructing a situation-model of the world, a self-model, and person models of others (Newen, 2015, 2018). In social situations these three processes are going on in parallel.

## Conclusion

To account for the rich phenomena of self-consciousness it is not sufficient to presuppose a brain and the neural processes which produce the phenomenology of self-consciousness. We need to presuppose *a real, embodied self*. In contrast to metaphysical theories of the self, the proposed pattern theory of the self is not a metaphysical substance or an entity with stable necessary and jointly sufficient conditions. The self is a flexible and varying embodied entity which we can only adequately account for *as an integrated pattern of characteristic features*. Furthermore, we have outlined how this pattern theory of the self fits into the predictive coding framework. All together, this enables us to accept an entity called a self within a naturalistic framework, and thereby we can account for a multiplicity of phenomena including long-term dispositions and autobiographical contents of a self.

## Author’s Note

This article is part of and supported by the Research Project “Situated Cognition” (DFG funding of the Research Training Group with grant number GRK 2185/1). Furthermore it is a product of the Humboldt awarded collaboration (Anneliese Maier Research Award of Prof. Gallagher) between myself and Shaun Gallagher since we are both developing versions of the pattern theory of self (Gallagher, 2013 and see the parallel publication in the same special issue, Gallagher and Daly, 2018). I would like to thank him, Somogy Varga and the members of my research group as well as two reviewers for very helpful feedback.

## Author Contributions

The author confirms being the sole contributor of this work and has approved it for publication.

## Conflict of Interest Statement

The author declares that the research was conducted in the absence of any commercial or financial relationships that could be construed as a potential conflict of interest.

## Footnotes

1 John Perry analyzes this immediate self-representation as being involved in two cases: either it is an agent-relative belief which is combined with the perspective of the particular agent where the agent need not have any concept, notion or idea of self; or it is a case of self-attached knowledge where the agent has to have an idea, notion or concept of self—Perry himself prefers to talk about ‘self-notions’ (Perry, 1998).

2 In such cases I would generalize the notion of a basic affective flow which involves conscious experiences, and speak of a minimal integration of basic self-directed information which does not need to involve any conscious experience, while self-directed information includes information about the cognitive system, its parts, and their relations processed by immediate self-representations.

3 To have a self does not presuppose the presence of consciousness, but only of an embodied cognitive system which is able to perceive and act in the world. Furthermore, it has to have immediate self-representations which are implemented as being about the system itself (as an agent) without any identification as an agent or a perceiver. Perhaps we will be able to construct robots which will implement a self, but in this article I focus on conscious human beings and set aside discussion of robots or non-human animals.

4 In philosophy, the discussion about the cognitive foundations of self-consciousness as an epistemic phenomenon and the discussion about the criteria which determine the identity of a person are virtually independent debates. This is systematically misleading, and is merely an accidental consequence of the history of the discussions. There are many advantages to ontologically identifying self and person, even though the terms highlight different epistemic or pragmatic dimensions (like modes of presentation) of the same entity. This leaves room for the claim that some embodied selves may not be persons, although – if this is so – this is not due to an ontological difference but to the fact that core practical features of the embodied self may not be implemented or respected in the relevant social environment.

5 There are multiple anti-realist positions in the literature which we do not need to consider since they are not naturalistic. Furthermore, one may reply that Metzinger could argue that there are different kinds of reality, for example natural and social realities. But, firstly, Metzinger does not distinguish different kinds of reality; and secondly, if such a distinction could be established, it would undermine Metzinger’s move from naturalistic constructivism to anti-realism. In his writings the ontological dimension of his claim is pretty clear until recently when he seems to constrain his ‘no self’-claim to a ‘methodological anti-realism’ with the moderate claim that “nothing in the scientific investigation of self-consciousness commits us to assume the existence of individual selves” (Metzinger, 2011, p. 285). Against this moderate claim, this article argues for an explanatory advantage of presupposing an embodied self.

6 This systematization is in its general structure parallel to the one which I developed for emotions (Newen et al., 2015). Inspired by my pattern theory of emotions, Gallagher has developed a first version of a pattern theory of self (Gallagher, 2013). The account I develop here bears some differences from Gallagher’s view, e.g., he includes extended aspects like physical pieces of property such as clothes, homes, and various things that we own, technologies we use, and institutions we belong to. According to my view, the embodied self is the primary self which manifests in biological instincts of self-care and self-preservation drives. But mainly we are sailing on the same boat.

7 It is worth highlighting again that the process that anchors the integrated pattern of characteristic features in the embodied self is realized by immediately self-related information such that the system the information is about cannot be misidentified because there is no identification involved. It is just a specific kind of information processing that makes it about the system. Vosgerau (2009) describes such a basic self-directed information processing as basic self-awareness. It can ontogenetically unfold into different types of self-consciousness (Newen and Vogeley, 2003).

8 I owe this terminological suggestion to Jennifer Windt.

9 In a recent article Metzinger discusses certain types of embodiment (Metzinger, 2014). But these are understood as embodied cognitive systems but not as an embodied self. Minimal phenomenal self-consciousness is only realized on a 3rd order embodiment which enables a cognitive system to establish a self-model and the self remains identical with the content of the self-model. Embodiment is only appreciated as a tool to develop a self-model.

10 It would go beyond the scope of this article to spell out the notion of accessibility. For the argument made here it is sufficient to accept that there are paradigmatic cases of easy access to autobiographic information in a situation (one’s name, family members, one’s last major birthday event), in contrast to very hard-to-access self-information (e.g., events which happened to you in your 2nd year of life or how active your own specific brain areas are). The latter can of course nowadays be made accessible with brain imaging techniques, but this is not something that is easily available.

11 J. M. Windt delivers a highly informed description about dreams and argues that we need to consider the actual bodily movement during sleep to adequately account for the dream experience. It is not sufficient to focus on the brain only: “Yet I argue that to understand bodily experience in dreams it is in fact crucial to look beyond the brain to real-body movements and sensations from the physical body during sleep” (Windt, 2018a, p. 20). This needs empirical evidence but seems at least to be a fruitful working hypothesis (Windt, 2018b).
